# Improved Tropical Forest Management for Carbon Retention

**DOI:** 10.1371/journal.pbio.0060166

**Published:** 2008-07-15

**Authors:** Francis E Putz, Pieter A Zuidema, Michelle A Pinard, Rene G. A Boot, Jeffrey A Sayer, Douglas Sheil, Plinio Sist, Elias, Jerome K Vanclay

## Abstract

Using reduced-impact timber-harvesting practices in legally logged tropical forests would reduce global carbon emissions by 0.16 Gt/year at a modest cost and with little risk of "leakage" (increased carbon emissions elsewhere).

Negotiations leading up to an international climate change agreement to replace the Kyoto Protocol in 2012 have included consideration of reduced emissions due to deforestation and degradation (REDD). This option has figured prominently in the “road map” toward such an agreement that was agreed upon during the 13th Conference of Parties to the United Nations Framework Convention on Climate Change held in Bali in December 2007 (http://www.unfccc.int/). Most ongoing discussions of REDD focus on tropical deforestation, while the potential carbon saving from reduced forest degradation is mostly disregarded [1,2]. Given that carbon losses due to degradation could be of the same magnitude as those from deforestation, this disregard is worrisome [3,4]. We show here that substantial reductions of global CO_2_ emissions can be achieved by improving forest management in the tropics, and argue that this cost-effective approach to mitigation should be included in the new climate change agreement.

Worldwide, a total area of 350 million hectares of tropical moist forests is designated as production forest [5], about a quarter of which is managed by rural communities and indigenous people [6]. These forests are mainly exploited for timber, and given growing timber demand and increased forest access, logging is likely to expand. Due to the high diversity of natural forests and limited markets for the timber of most tree species, loggers usually harvest only one to 20 trees per hectare. Unfortunately, for every tree logged in this selective manner, some ten to 20 others are severely damaged by untrained fellers and machine operators working without the aid of detailed maps or supervision (Protocol S1). Numerous studies have demonstrated that with appropriate harvest planning of log extraction paths, coupled with worker training in directional felling, 50% or more of this collateral damage can be avoided (Protocol S1). Implementing these basic reduced-impact logging techniques, as we show below, could substantially reduce global carbon emissions from forest degradation.

We illustrate the carbon benefits of improved forest management with a large-scale, long-term study in Malaysia (Protocol S1). In forests subjected to conventional logging, carbon emissions were over 100 tons per hectare (t ha^−1^) (Figure 1). In contrast, where improved harvesting practices were used, these initial losses were much lower, mainly due to reduced collateral damage. After 30 years, the typical period after which loggers are allowed to return to an area for the next harvest, carbon stocks in forests with improved management are predicted to be at least 30 t ha^−1^ higher than those in conventionally logged forests [7], and are probably much higher in practice. In a similar study in Amazonian Brazil, where forests are logged much less intensively [8], the benefit of improved timber harvesting practices was estimated to be 7 t C ha^−1^. In both cases, improved management reduced carbon emissions by approximately 30%, relative to conventional logging.

**Figure 1:**
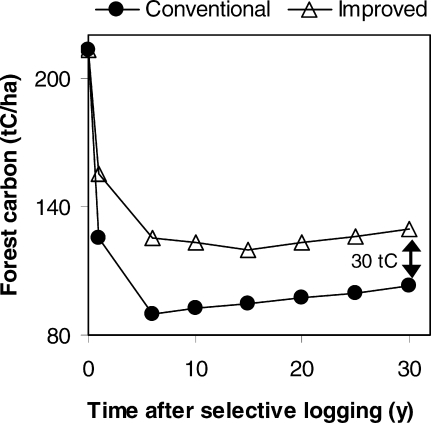
Substantial Reductions in Carbon Loss from Improved Forest Management at Scales of a Hectare of Malaysian Forest (Protocol S1)

The potential global contribution of improved tropical forest management to carbon retention is substantial. Using information on intensities and intervals of logging, area of production forest [5], and the above figures on carbon loss, we estimated the global consequences of improved tropical forest management (Protocol S1). We stress that this estimate is conservative, insofar as we lacked information to scale up the carbon-retention effects of some practices in improved forest management (improved road planning, bridge construction, stream protection). Also, our estimate of the production forest area most likely under-represents the actual area where logging takes place.

Use of improved timber harvesting practices in the tropical forests designated for logging would retain at least 0.16 gigatons of carbon per year (Gt C y ^−1^) (Figure 2). Most of the emission reductions from improved forestry will be from the more intensively logged forests in Asia, where emissions are largest. This reduced annual rate of carbon emissions is substantial. For comparison, the total amount of carbon emitted due to tropical deforestation is estimated to be 1.5 Gt y ^−1^ (or 20% of global anthropogenic emissions [1]). Thus, the potential for emission reductions through improved forest management is at least 10% of that obtainable by curbing tropical deforestation.

**Figure 2:**
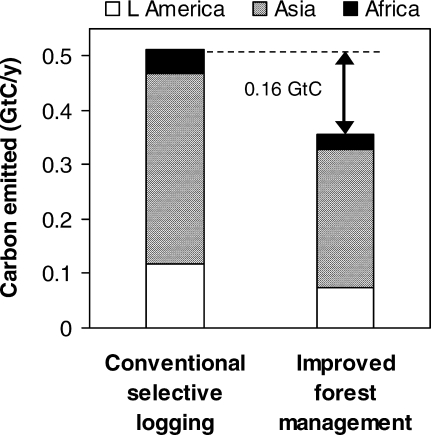
Annual Reductions in Global Carbon Emissions that Would Result from Adoption of Improved Tropical Forest Management Practices (Protocol S1)

Programs promoting global reductions in carbon emissions through improved forest management should easily satisfy the United Nations Framework Convention on Climate Change criteria for biomass projects (http://www.unfccc.int/). Given that improved forest management is currently practiced in less than 5% of tropical forests [5], the additionality criterion (i.e., the requirement that the intervention have direct impacts on carbon emissions relative to the baseline) seems easily satisfied if carbon policy-motivated interventions actually result in changes in timber harvesting practices. In regard to “permanence” of the sequestered carbon (i.e., the time period over which the carbon is retained), the long intervals between timber harvests in well-managed forests guarantee that the benefits of protecting trees from avoidable damage are lasting. And, given that timber harvests under improved management continue at the same intensity and with similar or even higher financial yields as those with more destructive conventional logging, there is little threat of loggers employing their destructive practices elsewhere and hence causing “leakage” of the carbon benefits (i.e., there is no incentive from the intervention for participants to increase carbon emissions elsewhere).

During the negotiations leading to the Kyoto Protocol, improved forest management practices were dropped from consideration due to concerns about the feasibility and costs of monitoring. Since that time, numerous field studies have demonstrated that forest carbon stocks can be measured with fair precision [8,9]. Of at least equal importance are the rapid recent improvements in remote sensing methodologies, which should soon make it feasible to directly estimate forest carbon stocks from space (Protocol S1) [4]. Furthermore, the costs of monitoring forest carbon stocks and fluxes, both on the ground and through remote sensing, can be shared by the responsible government agencies, forest product certifiers, and REDD credit programs. Clearly, if REDD provides a true and strong economic incentive to improve timber harvesting in the tropics, it will be cost-effective to solve any remaining technical difficulties.

In addition to increased carbon retention, there are important other benefits from improving forest management. For example, minimizing canopy opening allows forest flammability to decrease and shade-requiring wildlife and plants to continue to thrive. Also, future timber yields are enhanced when fewer of the valuable trees are damaged and when careful planning of roads and trails minimizes erosion and maintains watershed functions. Finally, substantial occupational health and safety benefits can be achieved from training workers in one of the world's most dangerous professions.

Incentives to retain more forest carbon through improved management would represent a big step toward sustainability in the vast area of tropical forests outside protected sites. Although many details on measuring, monitoring, and compensating carbon sequestering by individuals, companies, communities, and governments need to be sorted out, reducing emissions of greenhouse gases from tropical forest degradation should be given a high priority in negotiations leading up to the new climate change agreement to be formulated in Copenhagen in 2009.

## Supporting Information

Protocol S1 Background Information on Reduced-Impact Logging and Calculations of Carbon Emissions (46 KB DOC).

## Abbreviations

Gt C y ^−1^: gigatons of carbon per year
REDD: reduced emissions due to deforestation and degradation
tC ha ^−1^: tons of carbon per hectare
