# Supplementary material for: Improved Tropical Forest Management for Carbon Retention
Source: PLoS Biol. 2008 Jul 15;6(7):e166. doi: 10.1371/journal.pbio.0060166 (PMC2459208; doi:10.1371/journal.pbio.0060166)
Supplement: Supplementary file 1 [file pbio.0060166.sd001.doc]

**Protocol S1**

**Background information on Reduced Impact Logging and calculations of carbon emissions**

# Improved Tropical Forest Management for Carbon Retention

FE Putz, PA Zuidema, MA Pinard, RGA Boot, JA Sayer, D Sheil, P Sist, Elias, JK Vanclay

**Improved forest management: from conventional logging (CL) to reduced-impact logging (RIL)**

Reduced-impact logging (RIL) can be defined as intensively planned and carefully controlled timber harvesting conducted by trained workers in ways that minimize the deleterious impacts of logging (S1). RIL in the tropics has a long history but benefited from advancements in Australia in the 1980s and wide dissemination of basic guidelines by FAO in the 1990s (S2) as well as by development of regional and national codes-of-logging-practice. Table S1 gives an overview of the major techniques included in RIL, and their effects on trees, forests, and carbon.

Table S1. A partial list of practices customary included in reduced-impact logging guidelines and their effects on forests and carbon retention (S1-S6).

| Practice | Objective | Effect on forest^a^ | Effect on carbon retention | Included in our calculations? |
| --- | --- | --- | --- | --- |
| Planning of log landings | Reduce size and number of log landings | Substantial | Positive | No^b^ |
| Planning of roads | Reduce length and area of trails | Substantial | Positive | No^b^ |
| Construction of bridges, water culverts | Reduce water impoundments | Moderate | Positive | No^b^ |
| Planning of skid trails | Reduce soil and tree damage | Large | Positive | Yes |
| Marking of future crop trees | Reduce tree mortality and damage | Substantial / Large | Positive | Yes |
| Directional felling | Reduce tree damage and increase volume recovery | Substantial | Positive | Yes |
| Liana cutting | Reduce collateral damage and gap size | Substantial | Positive | Yes |

^a^ Categories of effect: Moderate <25%; Substantial 25-50%; Large >50%

^b^ Carbon retention effects can be large, if logging practices are poor (S7)

**The effects of RIL on forests and carbon**

Research on RIL has flourished over the past two decades, with more than 200 publications on the topic in many countries located in all three major tropical forest areas. RIL has been particularly effective in reducing tree mortality and damage (Table S1). By reducing damage to the forest, the most commonly used RIL practices have positive effects on carbon retention. In addition, worker training substantially reduces risks, which is relevant insofar as the International Labour Organization reports that logging is among the world’s most dangerous professions (S8).

**Recent improvements in remote sensing methods for estimating forest carbon stocks**

The use of laser altimetry (lidar) in combination with now traditional passive remote sensing methods heralds great strides towards direct measurement of forest carbon from space or airborne units (S9).

**Carbon emissions from conventional logging and RIL at the hectare scale**

We obtained carbon emissions from selectively logged forests from studies in Sabah, Malaysia (S5, S10) and Para, Brazil (S11-14). These studies are representative of the wide range of harvesting intensities and selective logging practices in the tropics. Commercial timber trees are abundant in SE Asian forests, leading to high logging intensities (~8-15 trees/ha, ~80-120 m^3^/ha), while in Latin America and Africa harvestable trees are scarcer and thus logging intensities are typically lower (~1-5 trees/ha, ~5-30 m^3^/ha).

Standing stocks of carbon in unlogged forests in both areas were calculated using field data on abundance and diameter of trees, allometric relations of trees, wood densities, abundance of non-woody plants, below-ground biomass, and biomass of woody debris and by assuming the carbon fraction of total dry mass to be 0.5. Resulting estimates of total carbon stocks in unlogged forests are 213 and 186 tC/ha for Malaysia (S5) and Brazil (S12), respectively, and are comparable to those from other studies (S14).

Both studies calculated carbon losses due to logging using field observations continuing up to 6 years after logging (timber volume removed, tree death, amount of woody debris, area cleared) (S5,S11,S13,S14), and simulation models of forest and carbon dynamics (using information on wood decay and tree growth rates) (S10,S12). The simulations allow estimation of carbon losses over several decades. In both studies, a comparison was made between conventional and reduced-impact logging (S10,S12).

Carbon losses due to selective logging at cycles (intervals) of 30 and 60 years decline substantially where reduced-impact logging (RIL) techniques are used (Table S2). Conventional logging leads to a carbon loss of as much as 50% in Malaysia whereas the losses estimated in Brazil (10-15%) are similar to those reported in other studies (5-20%, S16-18). RIL leads to substantially smaller loss of carbon, reducing these losses by 28-42% depending on location and logging interval. We report carbon consequences for 30 y because this is currently the most frequently prescribed logging cycle in tropical forests (S19), and for 60 y as this lapse in time between harvests is more likely to sustain timber yields and otherwise allow for better forest recovery (S2, S20-S22). The combination of applying RIL and lengthening the logging cycle from 30 y to 60 y would lead to much greater reductions in carbon loss than either change alone. For clarity, only the 30-y cycle results are presented in the main text, as these are closest to current practices, and do not alter profit margins.

Table S2. Carbon stocks, losses, and retention in unlogged and logged tropical rain forests in Malaysia and Brazil. Carbon consequences of conventional logging (CL) and reduced-impact logging (RIL) are compared.

|  | Sabah, Malaysia (S5,S9) | Para, Brazil (S10-13) |
| --- | --- | --- |
| Total carbon in unlogged forest (t ha^-1^) | 213 | 186 |
| Logging intensity (m^3^ ha^-1^) | 125 | 30 |
|  |  |  |
| Carbon loss and retention with 30-y logging cycles |  |  |
| - Loss from CL (t ha^-1^) | 108 | 19 |
| - Loss from RIL (t ha^-1^) | 78 | 12 |
| - Carbon retained due to RIL (t ha^-1^) | 30 | 7 |
|  |  |  |
| Carbon loss and retention with 60-y logging cycles |  |  |
| - Loss from CL (t ha^-1^) | 93 | 24 |
| - Loss from RIL (t ha^-1^) | 57 | 14 |
| - Carbon retained due to RIL (t ha^-1^) | 36 | 10 |

**Scaling up carbon emissions from conventional logging and RIL**

We used the information from the two studies in Table S2 to estimate global carbon retention resulting from improved forest management. We used the extent of tropical production forests reported by ITTO (S19) in the three continents with tropical forests (Asia, Meso- and South America, Africa) to estimate of the total area of forest under selective logging. We assumed a 30-y logging cycle as this best represents regulations and practice in Latin America, Africa, and Asia (S19). We then used the two available studies on carbon loss in selectively logged forests (Table S2) to convert carbon losses from hectare to continent scales. As logging intensities in Africa are comparable to those in Latin America, we used the results of the Brazil study there. We assumed that carbon impacts of logging at the continent-average intensity (30, 30, and 80 m^3^/ha for Americas, Africa and Asia, respectively) were adequately estimated from the two available studies (S5, S12). Given that logging damage increases little with harvests >80 m^3^/ha (S1), the estimates we use from in the Malaysia study (125 m^3^/ha harvested) are not expected to overestimate damage. We converted carbon loss values during one logging cycle (Table S2) to annual values by assuming that 1/30 of the area is logged annually, as is common in multi-cyclic silvicultural systems. Although the only two available studies on carbon impact of selective logging yield comparable results for carbon savings due to RIL as a proportion of carbon los through conventional logging, the extrapolated figures for global-scale effects should be interpreted cautiously. Nevertheless, our estimates of carbon retention are likely conservative if long-term recovery trajectories of RIL and conventional logging strongly differ due to arrested regeneration in the latter, compared to fast recovery in the former. Our estimates are also conservative insofar as they do not include the effects of forest clearance for poorly planned and unnecessary roads and log landings in forests with conventional logging (Table S1). Given that the data suggest that cutting cycles should be much longer than the typical 30 years, we include calculations of the carbon consequences forest management with 60-y cycles (S2, S20-23).

**References**

S1.	Putz FE, Sist P, Fredericksen TS, Dykstra D (2008) Reduced-impact logging:	challenges and opportunities. Forest Ecol Manage in press.

S2.	Dykstra DP, Heinrich R (1996) FAO Model Code of Forest Harvesting Practice. Food	and Agricultural Organization of the United Nations, Rome.

S3.	Sist P, Ferreira FN (2007) Sustainability of reduced-impact logging in the eastern	Amazon. Forest Ecol Manage 243: 199-209.

S4.	Sist P, Sheil D, Kartawinata K, Priyadi H (2003) Reduced-impact logging in	Indonesian Borneo: some results confirming the need for new silvicultural	prescriptions.	Forest Ecol Manage 179: 415-427.

S5.	Pinard MA, Putz FE (1996) Retaining forest biomass by reducing logging	damage.	Biotropica 172: 278-295.

S6.	Peña-Claros M, Peters EM, Justiniano MJ, Bongers F, Blate GM et al. (2008)	Regeneration of commercial trees species following silvicultural treatments in a	moist tropical forest. Forest Ecology and Management 255: 1283-1293.

S7.	Greenpeace, Carving up the Congo (2007). www.greenpeace.org/congoreport

S8.	International Labour Organisation (1990) Occupational safety and health in	forestry. Geneva, Switzerland.

S9.	Lefsky MA, Harding DJ, Keller M, Cohen WB, Carabajal CC et al. (2005)	Estimates of forest canopy height and aboveground biomass using ICEsat.	Geophys Res	Lett 32: L22S02.

S9.	Pinard MA., Cropper WP (2000) Simulated effects of logging on carbon storage in	dipterocarp forest. J Appl Ecol 37: 267-283.

S10.	Keller M, Palace, G. P. Asner, R. Pereira, J. N. M. Silva, Global Change Biology 10,	784 (2004).

S11.	Keller, MK, Asner GP, Silva N, Palace M (2004) Sustainability of selective	logging of upland forests in the Brazilian Amazon: carbon budgets and remote sensing	as tools for evaluating logging effects. In: Zarin DJ, Alavalapati JRR, Putz FE,	Schmink M, editors. Working forests in the Neotropics, New York: Columbia. pp. 41-63.

S12.	Palace M, Keller M, Asner GP, Silva JNM, Passos C. (2007) Necromass in	undisturbed and logged forests in the Brazilian Amazon. Forest Ecol Manage 238: 309-	318.

S13.	Keller M, Palace M, Hurtt G (2001) Keller M, Palace M, Hurtt G (2001). [Biomass	estimation in the Tapajos National Forest, Brazil - Examination of sampling and	allometric uncertainties](http://apps.isiknowledge.com/full_record.do?product=WOS&search_mode=GeneralSearch&qid=3&SID=3FmhMO1gmkeknokpcfp&page=1&doc=1). Forest Ecol Manage 154: 371-382.

S14.	Gibbs HK, Brown S, O Niles J, Foley JA (2007) [Monitoring and estimating	tropical forest carbon stocks: making REDD a reality](http://apps.isiknowledge.com/full_record.do?product=WOS&search_mode=GeneralSearch&qid=5&SID=3FmhMO1gmkeknokpcfp&page=1&doc=2). Environ Res Letters 2: 1-13.

S15.	Nepstad DC, Verissimo A, Alencar, A, Nobre, C, Lima E, et al. (1999). [Large-scale	impoverishment of Amazonian forests by logging and fire](http://apps.isiknowledge.com/full_record.do?product=WOS&search_mode=GeneralSearch&qid=6&SID=3FmhMO1gmkeknokpcfp&page=1&doc=1). Nature 398: 505-508.

S16.	Asner GP, Knapp, DE, Broadbent, EN, Oliveira PJC, Keller, M, Silva, JN. (2005).	[Selective logging in the Brazilian Amazon](http://apps.isiknowledge.com/full_record.do?product=WOS&search_mode=GeneralSearch&qid=8&SID=3FmhMO1gmkeknokpcfp&page=1&doc=1). Science 310: 480-482.

S17.	DeFries RS, Houghton, RA, Hansen, MC, Field, CB, Skole, D, Townshend, J. (2002).	[Carbon emissions from tropical deforestation and regrowth based on satellite	observations for the 1980s and 1990s](http://apps.isiknowledge.com/full_record.do?product=WOS&search_mode=GeneralSearch&qid=9&SID=3FmhMO1gmkeknokpcfp&page=1&doc=4). Proc Nat Acad Sci U S A 99: 14256-14261.

S18.	ITTO, Status of tropical forest management 2005, International Tropical Timber	Organization. www.itto.or.jp

S19.	Sist P, Fimbel R, Nasi R, Sheil D, Chevallier M-H (2003) Towards sustainable	management of mixed dipterocarp forests of South East Asia: moving beyond	minimum diameter cutting limits. Environ. Cons. 30: 364-374.

S20	Sist P, Picard N, Gourlet-Fleury S (2003). [Sustainable cutting cycle and yields in a	lowland mixed dipterocarp forest of Borneo](http://apps.isiknowledge.com/full_record.do?product=WOS&search_mode=GeneralSearch&qid=10&SID=3FmhMO1gmkeknokpcfp&page=1&doc=1). Annals of Forest Science 60: 803-814.

S21	Karsenty A, Gourlet-Fleury S (2006) Assessing sustainability of logging practices	in the Congo Basin’s managed forests: The issue of commercial species recovery.	Ecol	Soc. 11, 26 [online] http://www.ecologyandsociety.org/vol11/iss1/art26/

S22	van Gardingen, PR, Do Valle D, Thompson I (2006) Evaluation of yield	regulation	options for primary forest in Tapajos National Forest, Brazil. Forest Ecol Manage	231:	184-195.
